# Morphological characterization of *Iris hymenospatha* and *Iris histrio* populations in Iran: implications for conservation and breeding

**DOI:** 10.3389/fpls.2024.1305240

**Published:** 2024-05-28

**Authors:** Iman Rohollahi, Amir Mohammad Naji, J. Ryan Stewart, Rozita Kamrani

**Affiliations:** ^1^ Department of Horticulture Science, Faculty of Agriculture, Shahed University, Tehran, Iran; ^2^ Department of Agronomy and Plant Breeding, Faculty of Agriculture, Shahed University, Tehran, Iran; ^3^ Department of Plant and Wildlife Sciences, Brigham Young University, Provo, UT, United States; ^4^ Department of Horticulture Science, Shahed University, Tehran, Iran

**Keywords:** *Iris*, morphological, natural conditions, native, populations, variability

## Abstract

The native populations of *Iris hymenospatha* and *Iris histrio*, two endangered bulbous species within the large *Iris* genus in Iridaceae, are threatened with extinction due to mining and other industrial activities in their natural habitats in Central Asia, including Iran. These species not only have a significant economic impact on the global horticultural industry due to their versatility and attractive phenotypic traits, but also have significant ecological value that necessitates their conservation. In this study, we examined the morphological and functional diversity between individuals within these two species, which exhibit high tolerance to environmental stresses. Our study examined 10 populations of *I. hymenospatha* and two populations of *I. histrio* based on bulb, flower, and leaf characteristics throughout Iran. We recognized a gradation of five different leaf shapes among *I. hymenospatha* populations with significant differences between some populations, including “Arak-Khomain” and “Arak-Gerdo”. The “Jaro”, “Natanz-Karkas”, “Ardestan-Taleghan”, “Arak-Rahjerd”, “Arak-Gerdo”, “Ganjnameh”, and “Abas-Abad” populations of *I. hymenospatha* displayed maximal values in leaf width, stem diameter under flower, crown diameter, flower number, leaf number, and bulb diameter. The *I. histrio* “Velian” population had a significantly larger flower size, a longer stem length, a larger style width, a longer flowering date, and a higher plant height compared to the “Ganjnameh” population of *I. histrio*. Such characteristics of both species make them remarkable ornamental plants. Our study also revealed that *I. hymenospatha* populations grow on different soils and elevations and have the ability to adapt to different growing conditions. Given the threats they face, conservation through horticultural selection and propagation offers a viable conservation strategy for both species. This approach not only preserves the genetic diversity of these species, but also enables their further contribution to the horticultural industry.

## 1 Introduction

*Iris sensu lato*, a large genus within Iridaceae, as delineated by Mavrodiev et al. (2014), comprises 260–300 species worldwide, which are distributed throughout the Northern Hemisphere (Lawrence, 1953; Mathew, 1989, 2000; Weber et al., 2020). While some *Iris* species naturally occur in mesic and wetland environments, the majority of species grow in arid, semi-arid, or dry montane environments (Roguz et al., 2020). Species within the genus exhibit a wide variety of flower color, including a range of hues of blue, violet, yellow, orange, and even black (Roguz et al., 2020). Such characteristics make *Iris* widely popular as a perennial garden plant. Taxonomists consider between 21 and 26 *Iris* species to be native to Iran (Azimi et al., 2018). Indeed, few reports exist regarding the *Iris* species native to Iran. *Iris hymenospatha* B. Mathew & Wendelbo and *Iris histrio* Rchb.f. naturally occur throughout Iran on dry mountain slopes (Ghahreman, 1984). The native range of *I. hymenospatha* extends through Iran and the Kurdistan region of Iraq (Wendelbo, 1977), whereas that of *Iris histrio* stretches from Iran to Turkey (RBG, 2024).

*Iris histrio* and *I. hymenospatha* have long been recognized as members of two different groups of *Iris*, although the taxonomic levels and names given to these two groups have been disputed (C. Wilson, personal communication). We are following the *Iris* classification scheme of Wilson (2011) in recognizing *I. histrio* in *Iris* section *Reticulata* Dykes, housed in *Iris* subgenus *Xiphium* Spach, and *Iris hymenospatha* in the *Scorpiris* Spach. section of the *Iris* subgenus *Scorpiris* Spach.

Species within the *Iris* genus have a sizable economic impact on the global horticulture industry (Khatib et al., 2022). Several *Iris* species and cultivars can be commonly found in gardens and landscapes throughout the world due to their large, colorful flowers and visually appealing linear leaves (Xu et al., 2019). The fragrant violet-like nature of the floral blooms contributes to their ornamental value (Amin et al., 2021). Beyond their horticultural value, several species are utilized in different traditional medicines to treat inflammation, cancer, and bacterial and viral infections (Amin et al., 2018). Essential oils extracted from *I. hymenospatha* flowers and leaves exhibited relatively high antifungal activity against three human-pathogenic fungal strains (*Aspergillus niger* 2CA936, *Aspergillus flavus* NRRL3357, and *Candida albicans* ATCC1024) (Khatib et al., 2022).

Plant breeding often involves phenotypic and genotypic characterization to capitalize upon genetic variation to select for desirable traits (Xu et al., 2019; Abenavoli et al., 2021). Understanding the genetic diversity and relationships among plant species, cultivars, and varieties underscores the importance of availability of wild genetic resources for breeding improvement efforts (Azimi et al., 2019), especially for those species that are rare or at risk of extinction (Perrino and Calabrese, 2018; Perrino and Wagensommer, 2022), or belonging to problematic taxonomic groups (Wagensommer et al., 2016), which are not favored by intensive agriculture (Calabrese et al., 2015). In theory, when high genotypic diversity occurs in plant populations, one or more individuals will likely be well adapted to various forms of environmental stress (Reynolds et al., 2012). Microenvironments, which act as powerful selective forces, are more likely to be found in close proximity rather than at larger distances (Reynolds et al., 2012).

*Iris hymenospatha* and *I. histrio* populate various habitats, including dry, rocky areas; semi-deserts; and wetlands (Hussain et al., 2020). Azimi et al. (2016) studied diversity within native Iranian *Iris* species using morphological traits and identified economically significant quantitative traits, including flower size, outer and inner tepal width, and leaf width. These traits may be considered in breeding programs, with the outer and inner tepal width and inner tepal length being important components of the flower (Azimi et al., 2016). An analysis of *Iris* × *germanica* L. hybrids found that the highest coefficient of variation among *Iris* species is linked to several traits, including flower height, diameter, filament length, seed diameter, and leaf number, with high heritability values for plant height, fall width, fall length, flower size, and leaf width (Azimi et al., 2018). Asgari et al. (2022) studied Iran’s endemic *Iris* species and identified four ecotypes of *I. hymenospatha* species in west-central Iran as highly suitable accessions for further selection due to their height, large flower size, and high durability.

These bulbous species generally bloom in late winter and early spring. *Iris hymenospatha* and *I. histrio* typically bloom at the end of March and the beginning of April. From a horticultural perspective, the springtime flowering habit of these species is ideal for commercial growers to market and sell this species. Moreover, these bulbous *Iris* species can be easily propagated through vegetative divisions (Kamenetsky and Okubo, 2012). According to Hassani et al. (2008), climatic conditions, grazing, and mining activities (Gholamzade Natanzi, 2023) are key elements that influence species composition and rangeland biodiversity in semi-arid ecosystems in Iran. In their native localities, *I. hymenospatha* and *I. histrio* are among the first plant species to bloom in early spring. This makes them particularly vulnerable to overgrazing by livestock, which can lead to a significant decline in the growth vigor and reproductive ability of these species. Overgrazing is a major problem for the conservation of these species, as it can lead to the destruction of their habitat and the loss of biodiversity (Farahmand and Farzad Nazari, 2015). Moreover, owing to the narrow ecological habit of *I. hymenospatha* and *I. histrio*, specimens of both species appear to naturally occur singly or in small populations (I. Rohollali, personal observation).

Horticulture plays an important role in conserving biodiversity and promoting sustainable development (Kumar, 2014). Little, however, appears to be known regarding the morphological diversity of *I. hymenospatha* and *I. histrio* within their native range. Therefore, 100 native specimens of *I. hymenospatha* and 20 accessions of *I. histrio* from native populations were identified, collected, and characterized from 12 sites (**Table 1**). In this study, we tested the hypothesis that accessions from both species from different habitats exhibit significant diversity in their leaf, bulb, and flower characteristics. Consequently, we examined morphological variability among populations to identify the most useful phenotypic variables for discrimination within populations that could serve as a genetic resource for breeding programs. This study appears to be the first to characterize *I. hymenospatha* and *I. histrio* populations based on morphological traits within their native range in Iran.

**Table 1:** Distribution and identification of native populations of *Iris hymenospatha* and *Iris histrio* across Iran: detailed geographical data and corresponding species found in each area.

No.	Genus and species	Province	Region	Accessions per population	Latitude	Longitude	Altitude (m)	Total precipitation (mm) average month	Max/Min temperature
1	Iris hymenospatha	Alborz	Jaro	10	35° 40.822′	50° 33.186′	1,655	41	33.9/−4.8
2		Isfahan	Natanz-Karkas	10	33° 30.120′	51° 50.121′	2,197	30	37.3/0.7
3		Isfahan	Ardestan-Taleghan	10	33° 16.374′	52° 11.756′	2,036	19	44/10
4		Markazi	Arak-Rahjerd	10	34° 22.140′	50° 21.477′	1,767	46	34.5/−3.9
5		Markazi	Arak-Khomain	10	33° 57.956′	49° 52.358′	1,802	39	34.1/−3.8
6		Markazi	Arak-Gerdo	10	34° 03.351′	49° 41.005′	1,957	45	34.3/3.9
7		Hamedan	Ganjnameh	10	34° 45.430′	48° 26.290′	2,209	71	32.4/−3.4
8		Hamedan	Abas-abad	10	34° 46.560′	48° 28.263′	2,066	71	32.4/−3.4
9		Fars	Arsanjan	10	29° 52′ 40.3″	53° 19′ 18.2″	1,700	21	35.5/5.1
10		Isfahan	Ardestan-Mishab	10	33° 11′ 12.5″	52° 33′ 14.7″	2,100	–	44/10
11	Iris histrio	Hamedan	Ganjnameh	10	34° 45.441′	48° 26.272′	2,212	–	32.4/−3.4
12		Alborz	Velian	10	36° 02.444′	50° 50.036′	1,988	47	33.9/−4.8

## 2 Materials and methods

To determine the morphological diversity of *I. hymenospatha* and *I. histrio* populations within their native range, we evaluated 30 morphological traits (**Table 2**). We collected 10 accessions per population from each of the 10 populations of *I. hymenospatha* and from 2 populations of *I. histrio*, totaling 120 accessions, along the eastern edge of the Zagros Mountains in western Iran (**Table 1**). To clarify, there were 100 accessions of *I. hymenospatha* and 20 accessions of *I. histrio*. Although native to the region, *I. histrio* populations appear to be few and infrequent in number. Accessions were identified using morphological keys and taxonomic descriptions of the species (Wendelbo, 1977; Azimi et al., 2019). The geographical data of the study area are presented in **Table 1**. We analyzed the morphological variance of *I. hymenospatha* and *I. histrio* populations to characterize their phenotypic trait diversity (**Table 3**).

**Table 2:** List of morphological characters of *Iris hymenospatha* and *Iris histrio* studied and their corresponding abbreviations.

No.	Character	Abbreviation	Unit	No.	Character	Abbreviation	Unit
1	Bulb diameter	BD	mm	16	Standard (petal) length	SL	mm
2	Bulb length	BL	mm	17	Standard (petal) width	SW	mm
3	Leaf number	LN	–	18	Style arm width	SA	mm
4	Leaf width	LW	mm	19	Style crest length	SCL	mm
5	Plant length	PL	cm	20	Style crest width	SCW	mm
6	Crown diameter	CD	mm	21	Anther length	AL	mm
7	Stem length under flower	SLF	cm	22	Flower color	FC	Code
	Stem diameter under flower	SDF	mm	23	Inside flower color	IFC	Code
9	Flower number	FN	Number	24	Flowering date	FD	Code
10	Flower size	FS	mm	25	Floral scent	FS	Code
11	Peduncle length	PL	mm	26	Bulb tunic type	BTT	Code
12	Peduncle diameter	PD	mm	27	Leaf shape	LS	Code
13	Spathe (covering ovary)	S (CO)	mm	28	Bulb tunic color	BTC	Code
14	Falls (sepal) width	FW	mm	29	Leaf margin color	LMC	Code
15	Signal width	SW	mm	30	Bulb tunic density	BTD	Code

**Table 3:** Comprehensive analysis of morphological variance (AMOVA) in *Iris hymenospatha* and *Iris histrio* populations.

S.O.V	df	Flower size	Peduncle length	Peduncle diameter	Spathe (covering ovary)	Falls (sepal) width	Signal width	Standard (petal) length	Standard (petal) width	Style arm	Style crest length	Style crest width	Anther length
Between pop	7	342.17^**^	412.42^**^	4.74^**^	49.43^*^	9.93^**^	3.92^**^	2401.92^**^	78.5^**^	13.1^**^	9.71^**^	3.32^**^	241.29^**^
Error	58	26.86	36.76	0.67	21.58	2.95	0.56	8.73	94.11	0.77	1.69	0.54	7.51

*, ** Significant difference in 5%, 1% level respectively.

### 2.1 Morphological evaluation

Thirty floral and morphological traits, such as quantitative and qualitative characteristics of leaves, bulb, and flower, were measured (**Tables 2**, **4**). Among the *I. hymenospatha* and *I. histrio* populations, flower characteristics were recorded for only 6 of the 10 populations of *I. hymenospatha* and the 2 populations of *I. histrio* due to differences in their respective flowering times in the wild (**Table 5**). Measurements of all morphological traits were taken in the field during the peak of flowering season (mid-February to early March) in 2022. The populations are relatively clustered, with up to two accessions per square meter, with there being several dozen plants per population. When sampling in the field, population boundaries were clearly recognizable. Accessions were defined as clumps (ramets) of leaf fans spaced more than 30–100 m apart. All accessions were evaluated in each population. Quantitative traits such as leaf width, plant length, flower size, fall width, standard length, anther length, bulb length, bulb diameter, and stem length were evaluated using a digital caliper (**Table 2**). We also measured leaf number and flower number. In addition, qualitative characteristics, such as flower scent, flower color, inner flower color, flowering date, bulb tunic type, leaf shape, bulb tunic color, leaf margin color, and bulb tunic density, were surveyed based on rating, scoring, and coding (**Table 4**).

**Table 4:** Qualitative trait descriptors used in the study of *Iris hymenospatha* and *Iris histrio* populations.

Character	Code and state						
	1	2	3	4	5	6	7
Flower color	Mint cream	Sky blue	Pale turquoise	Light sky blue	Lavender	Lilac	Light steel blue
Inside flower color	Yellow-blue	Yellow-green	Yellow-white	White-blue-yellow	White-purple	Purple-yellow	White-purple-yellow
Flowering date	Middle of March	Late March	Early April	–	–	–	–
Floral scent	Little	No scent	High	–	–	–	–
Bulb tunic type	Papery	Leathery	–	–	–	–	–
Leaf shape	Short erect leaves	Middle erect leaves	Short sickle-shaped leaves	Long erect leaves	Long sickle-shaped leaves		
Bulb tunic color	Cream	Light brown	Dark brown	–	–	–	–
Leaf margin color (LMC)	With LMC	Without LMC	–	–	–	–	–
Bulb tunic density	Little	Moderate	High	–	–	–	–

**Table 5:** Detailed morphological characteristics of *Iris hymenospatha* and *Iris histrio* flowers: analysis of flower size, spathe, standard (petal), style crest, and anther length across populations.

No.**		Flower size (mm)	Peduncle length (mm)	Peduncle diameter (mm)	Spathe (covering ovary) (mm)	Falls (sepal) width (mm)	Signal width (mm)	Standard (petal) length (mm)	Standard (petal) width (mm)	Style arm width (mm)	Style crest length (mm)	Style crest width (mm)	Anther length (mm)
1	*I. hymenospatha*	45.9 ± 1.7 c*	35.2 ± 1.9 a	4.9 ± 0.3 abc	19.0 ± 1.5 a	10.6 ± 0.5 ab	5.1 ± 0.2 a	4.0 ± 0.9 c	11.0 ± 0.4 a	8.7 ± 0.3 a	8.8 ± 0.4 b	4.7 ± 0.2 bc	18.9 ± 0.9 bc
2		47.0 c ± 1.7	20.8 ± 1.9 c	4.2 ± 0.3 bcd	18.1 ± 1.5 a	10.0 ± 0.5 bc	4.9 ± 0.2 bc	4.0 ± 0.9 c	10.7 ± 0.4 a	8.0 ± 0.3 a	8.7 ± 0.4 b	5.1 ± 0.2 a	21.6 ± 0.9 b
3		40.2 ± 2.1 d	21.7 ± 2.5 bc	3.9 ± 0.3 cd	18.8 ± 1.9 a	9.5 ± 0.7 abc	4.4 ± 0.3 bc	10.3 ± 1.2 b	4.0 ± 0.5 b	8.0 ± 0.4 ab	8.9 ± 0.5 b	4.2 ± 0.3 bc	22.3 ± 1.1 b
4		48.9 ± 3.0 cb	15.6 ± 3.5 cd	5.0 ± 0.5 abc	9.2 ± 2.7 b	10.0 ± 0.1 bc	5.0 ± 0.4 bc	12.7 ± 1.7 b	4.6 ± 0.7 b	7.5 ± 0.5 ab	8.7 ± 0.8 b	4.4 ± 0.4 abc	22.8 ± 1.6 b
7		47.0 ± 2.0 c	13.4 ± 2.31 d	5.1 ± 0.3 ab	15.5 ± 1.8 ab	11.5 ± 0.7 a	5.2 ± 0.3 a	12.1 ± 1.1 b	5.0 ± 0.5 b	8.3 ± 0.3 a	7.6 ± 0.5 b	4.9 ± 0.3 ab	20.5 ± 1 bc
8		45.5 ± 1.6 c	19.1 ± 1.8 cd	5.2 ± 0.2 a	15.8 ± 1.4 a	9.3 ± 0.5 bc	5 ± 0.2 ab	12.1 ± 0.9 b	4.2 ± 0.4 b	7.1 ± 0.3 b	8.0 ± 0.4 b	4.6 ± 0.2 ab	18.3 ± 0.8 c
11	*I. histrio*	52.7 ± 1.7 b	18.8 ± 1.9 cd	3.5 ± 0.3 d	17.7 ± 1.5 a	9.0 ± 0.5 bc	4.2 ± 0.2 b	41.8 ± 0.9a	4.8 ± 0.4 b	5.6 ± 0.3 c	8.50 ± 0.4 b	3.5 ± 0.2 c	30.5 ± 0.9 a
12		61.3 ± 1.6 a	27.9 ± 1.9 b	3.4 ± 0.3 d	19.7 ± 1.5 a	7.9 ± 0.5 c	3.2 ± 0.2 c	43.3 ± 0.9 a	4.7 ± 0.4 b	5.5 ± 0.3 c	11.0 ± 0.4 a	3.6 ± 0.2 c	31.3 ± 0.9 a

*Data in the columns marked with the same letter of the alphabet do not differ significantly at *p* ≤ 0.05. The heat map visually represents the relationships among the morphological characteristics of *I. hymenospatha*. Specifically, it illustrates the correlations between data columns labeled with the same letter, as demonstrated by the heat map showing relationships among columns marked with identical letters in the *I. hymenospatha* morphological characteristic data (red color = maximum value for each trait, yellow = moderate, and green = minimum value for each trait).

**Populations 5, 9, and 10 did not have any flowers.

### 2.2 Statistical analysis

**Figure 1:**
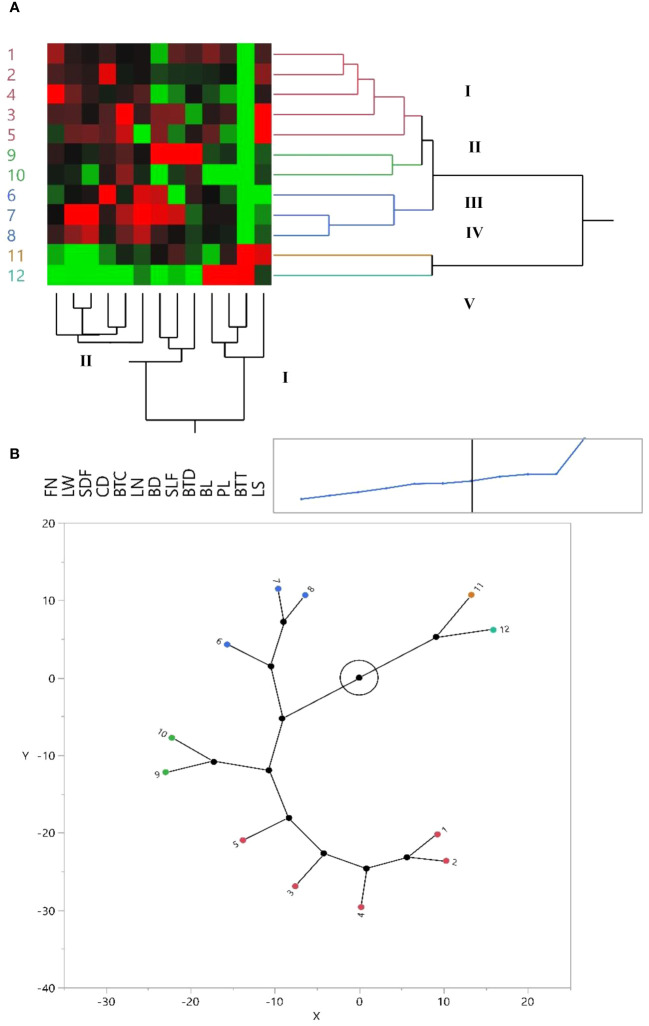
**(A)** Dendrogram and heat map derived from hierarchical clustered analysis of *Iris hymenospatha* in 10 populations and *Iris histrio* in 2 populations based on morphological traits. Red color = maximum value, black = moderate, and green = minimum value for each trait. See **Table 2** for the definition of trait abbreviations. Similarity: Euclidean distance. Method: Ward’s linkage. **(B)** Constellation plot of *I. hymenospatha* and *I. histrio* populations.

**Figure 2:**
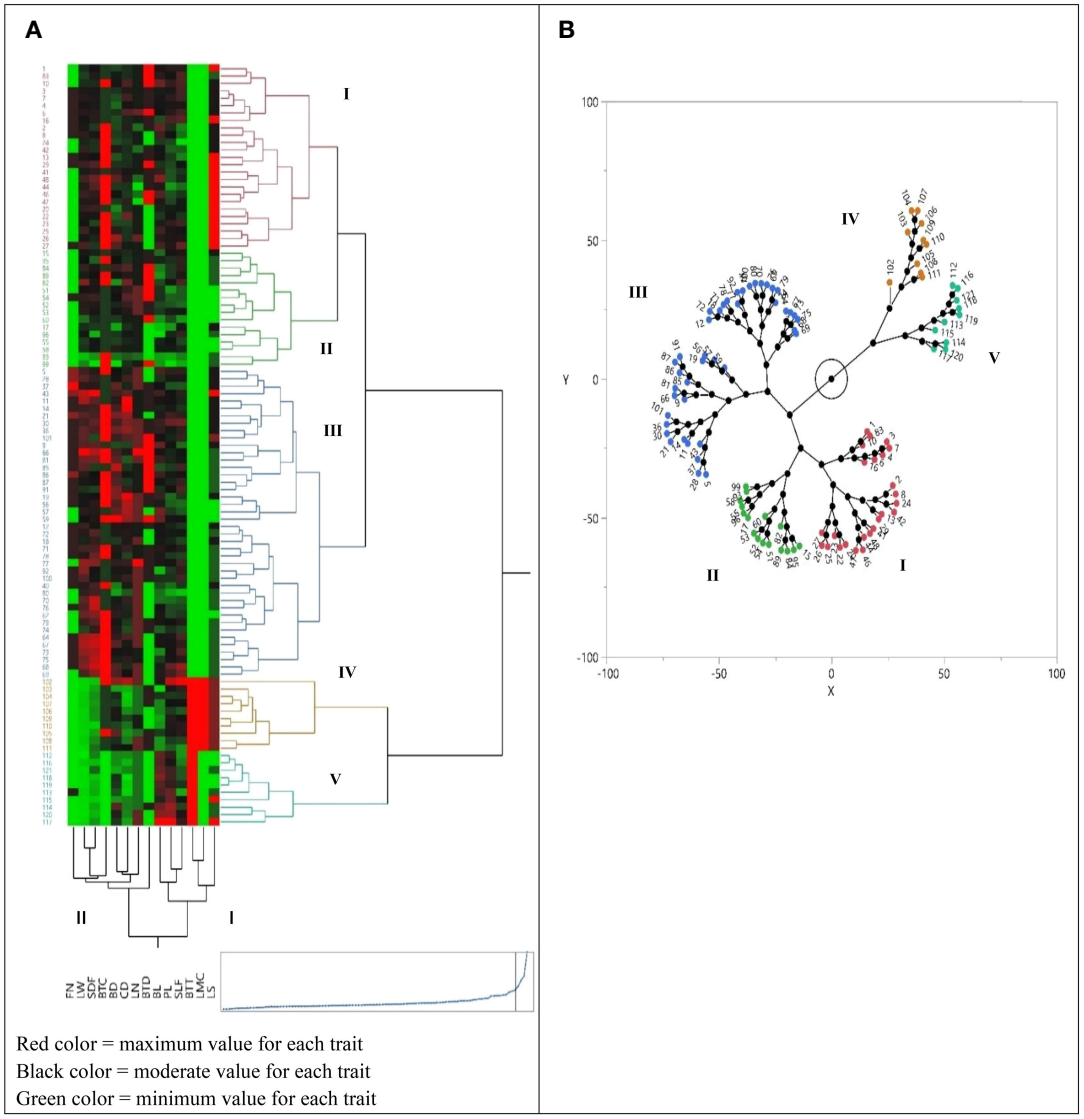
**(A)** Cluster analysis of 100 accessions of *Iris hymenospatha* belonging to 10 populations and 20 accessions of *Iris histrio* belonging to 2 populations based on morphological traits using Euclidean distances. Heat map shows the relationship between cluster analysis of morphological characteristic data. See **Table 2** for the definition of trait abbreviations. **(B)** Cluster analysis depicting the constellation lot of *I. hymenospatha* and *I. histrio* accessions.

**Figure 3:**
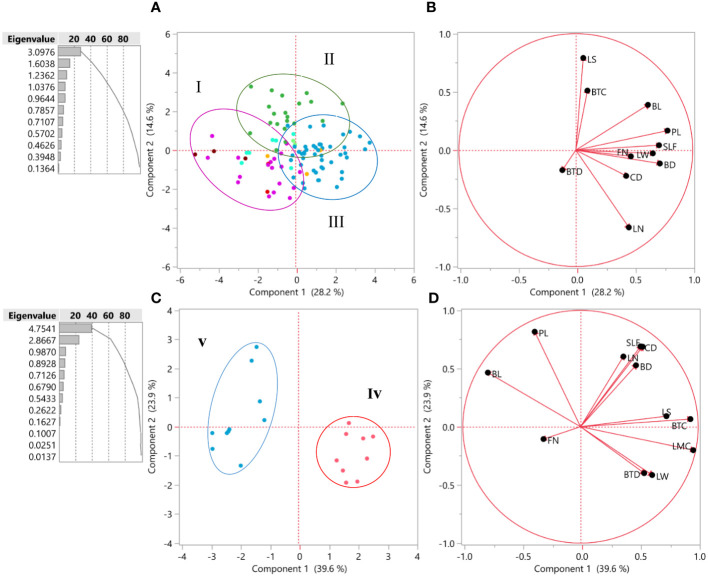
**(A)** Biplot for the first two principal components’ 100 accessions of *Iris hymenospatha* based on morphological traits. Population colors indicate the three groups identified through the PCA [I = purple, II = green, and III = blue]. **(B)** The most effective morphological traits based on the first two principal components. **(C)** Biplot for the first two principal components’ 20 accessions of *Iris histrio* based on morphological traits. Population colors indicate the two groups identified through the PCA [iv = red, v = blue]. **(D)** The most effective morphological traits based on the first two principal components. leaf number, flower number, crown diameter, stem diameter under flower, leaf width, and bulb tunic color.

The morphological properties were statistically analyzed by JMP Pro software version 13 (SAS Institute Inc. 2017. JMP^®^ 13 Consumer Research, Second Edition). Analyses of variance were performed to detect significant differences between populations with the means being compared using the Duncan’s multiple range tests. Cluster analysis was performed using the unweighted pair group method with an arithmetic average (UPGMA) using five qualitative and nine quantitative traits that we collected for every population (**Figures 1A, B**, **2A, B**). For qualitative traits, the statistical significance was assessed using a two-tailed non-parametric Kruskal–Wallis test. Relationships among the accessions were investigated by principal component analysis (PCA) using JMP Pro. Additionally, the scatter plot was made utilizing the first and second principal components (PC1/PC2) with JMP Pro. The biplot for the first two PCs showed consistent results for 100 accessions of *I. hymenospatha* (**Figure 3A**) and 20 accessions of *I. histrio* (**Figure 3C**) based on morphological traits. In addition, the most effective morphological traits were determined based on the first two PCs and are shown in **Figures 3B, D**.

Both species have been included in a single analysis to obtain a single figure for the cluster analysis for the specific purpose of visually summarizing the data. This approach was taken to simplify the presentation of the data and to avoid any potential confusion that could arise from presenting the data separately for each species. Additionally, *I. hymenospatha* and *I. histrio* were analyzed separately in PCA to better understand the differences between the two species.

## 3 Results

**Figure 4:**
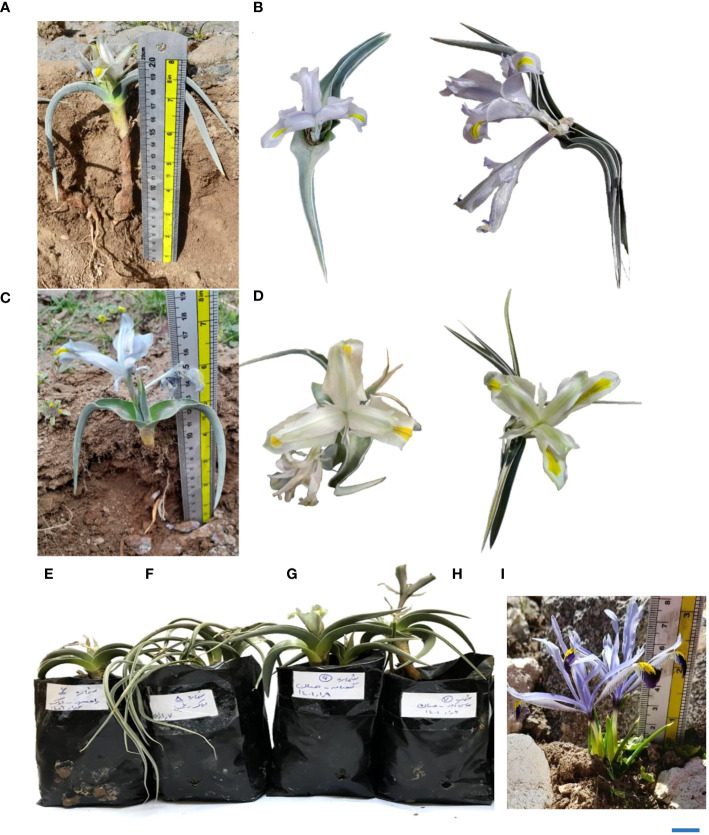
**(A)** Comparative display of flower and leaf shapes in various *Iris hymenospatha* and *Iris histrio* populations: detailed morphological differences in *I. hymenospatha* “Natanz-Karkas”, **(B, E)** *I. hymenospatha* “Arak-Rahjerd” (long erect leaf shape), **(C)** *I. hymenospatha* “Jaro”, **(D, H)** *I. hymenospatha* “Abas-abad” (middle erect leaf shape), **(F)** *I. hymenospatha* “Arak-khomain” (long sickle-shaped leaf shape), **(G)** *I. hymenospatha* “Ganjnameh” (long erect leaf shape), and **(I)** *I. histrio* (bar = 5 cm).

The use of morphological descriptors to characterize wild populations of *I. hymenospatha* and *I. histrio* reveals wide variation among most flower, bulb, and leaf morphological traits, such as flower size, peduncle length, flower number, bulb length, leaf number, leaf width, plant length, crown diameter, flower color, and leaf shape (**Tables 5**–**7**; **Figure 4**).

**Table 6:** Detailed examination of bulb and leaf morphological characteristics in *Iris hymenospatha* and *Iris histrio* populations.

No.	Genus and species	Flower number (mm)	Bulb diameter (mm)	Bulb length (mm)	Leaf number (mm)	Leaf width (mm)	Plant length (mm)	Crown diameter (mm)	Stem length under flower (mm)	Stem diameter under flower (mm)
1	I. hymenospatha	1.9 ± 0.2 ab*	16.5 ± 1 b	29.1 ± 1 b	3.2 ± 0.2 abcd	14.5 ± 1.1 bcd	18.3 ± 0.9 b	7.3 ± 0.4 ab	12.9 ± 0.7 a-d	9.8 ± 0.6 cd
2		1.7 ± 0.2 abc	17.5 ± 1 ab	25.0 ± 1 d	3.2 ± 0.2 abcd	14.9 ± 1.1 bcd	16.3 ± 0.9 bcd	8.0 ± 0.4 ab	11.7 ± 0.7 a-e	10.1 ± 0.6 cd
3		1.7 ± 0.2 abc	19.4 ± 1 ab	26.7 ± 1 bcd	3.3 ± 0.2 abc	16 ± 1.1 bc	18.3 ± 0.9 b	7.4 ± 0.4 ab	13.1 ± 0.7 abc	9.7 ± 0.6 cd
4		2.2 ± 6 2 a	16.3 ± 1 b	23.9 ± 1 de	3.4 ± 0.2 abc	16.9 ± 1.1 b	13.7 ± 0.9 de	6.8 ± 0.4 bc	11.0 ± 0.7 cde	10.4 ± 0.6 c
5		1.3 ± 0.2 cde	19.5 ± 1 ab	28.5 ± 1 bc	2.2 ± 0.2 e	16.7 ± 1.1 b	13.8 ± 0.9 de	7.5 ± 0.4 ab	11.1 ± 0.7 cde	12.1 ± 0.6 b
6		1.2 ± 0.2 cde	20 ± 1 a	25.8 ± 1 cd	3.7 ± 0.2 a	13.0 ± 1.1 cd	15.0 ± 5.0 cde	8.2 ± 0.4 a	10.4 ± 0.7 e	9.9 ± 0.6 cd
7		1.5 ± 0.2 bc	20.2 ± 1 a	26.3 ± 1 bcd	3.8 ± 0.2 a	20.8 ± 1.1 a	17.1 ± 7 8 bc	7.2 ± 0.4 ab	13.6 ± 0.7 ab	14.9 ± 0.6 a
8		1.6 ± 0.2 abc	18.8 ± 1 ab	25.5 ± 1 cd	3.6 ± 0.2 ab	17.6 ± 1.1 b	16.0 ± 0.9 bcd	6.8 ± 0.4 bc	12.2 ± 0.7 a-e	13.6 ± 0.9 ab
9		1.6 ± 0.2 abc	20.5 ± 0 a	24.0 ± 1 de	3.2 ± 0.2 abcd	13.2 ± 1.1 cd	15.6 ± 0.9 bcd	7.3 ± 0.4 ab	14.0 ± 0.7 a	8.4 ± 0.6 d
10		1.4 ± 0.2 bcd	16.1 ± 1 b	20.8 ± 1 f	3.0 ± 0.2 bcd	11.6 ± 1.1 d	12.8 ± 0.9 e	7.2 ± 0.4 ab	11.6 ± 0.7 b-e	5.1 ± 0.6 e
11	I. histrio	1.0 ± 0.2 de	18.4 ± 1 ab	21.6 ± 1 ef	2.8 ± 0.2 cde	2.5 ± 7 2 e	17.8 ± 0.9 b	5.8 ± 0.4 cd	12.7 ± 0.7 a-e	4.1 ± 0.6 e
12		0.8 ± 0.2 e	16.1 ± 1 b	32.1 ± 1 a	2.6 ± 0.2 de	2.5 ± 1.1 e	23.1 ± 0.9 a	5.0 ± 0.4 d	10.7 ± 0.7 de	4.0 ± 1 9 e

*Data in the columns marked with the same letter of the alphabet do not differ significantly at *p* ≤ 0.05.

The heat map visually represents the relationships among the morphological characteristics of *I. hymenospatha* and *I. histrio*. Specifically, it illustrates the correlations between the data columns that are labeled with the same letter (red color = maximum value for each trait, green = moderate, and blue = minimum value for each trait).

**Table 7:** Qualitative trait morphology and Kruskal–Wallis test results: A comparative study of *Iris hymenospatha* and *Iris histrio* populations.

Sample no.**		Mean of ranks								
		Flower color	Inside flower color	Flowering date	Floral scent	Bulb tunic type	Leaf shape	Bulb tunic color	Leaf margin color	Bulb tunic density
1	I. hymenospatha	42.2 abc*	23.1 c	10.5 b	32.5 b	51 b	79.2 ab	57.5 a	56 b	75.5 ab
2		24.3 bc	24.5 bc	30 ab	32.5 b	51 b	86.6 ab	52 ab	56 b	56.4 b
3		17.0 c	36.2 abc	30 ab	32.5 b	51 b	97.7 a	85 a	56 b	37.3 b
4		23.9 bc	26 abc	30 ab	32.5 b	51 b	75.5 abc	63 a	56 b	63.7 ab
5		0	0	0	0	51 b	97.7 a	79.5 a	56 b	68.2 ab
6		24.2 bc	24.5 abc	30 ab	32.5 b	51 b	9.5 d	46.5 ab	56 b	71.0 ab
7		28.5 bc	9 c	53 a	32.5 b	51 b	26.9 cd	78.8 a	56 b	45.5 b
8		67 a	23 c	53 a	32.5 b	51 b	38.5 bcd	70.5 a	56 b	52.7 b
9		0	0	0	0	51 b	38.5 bcd	68.5 a	56 b	106.5 a
10		0	0	0	0	51 b	44.5 bcde	74 a	56 b	75.5 ab
11	I. histrio	47.5 a	49 ab	53 a	32.5 b	111.5 a	98 a	46.5 ab	56 b	51.8 b
12		47.5 a	53 a	10.5 b	69.5 a	111.5 a	41.7 bcd	5.5 b	116.5 b	29.0 b
Kruskal Wallis chi-square		41.2	40.2	65	73	120	93.82	52.6	120	43.2
Significance		< 0.0001	< 0.0001	< 0.0001	< 0.0001	< 0.0001	< 0.0001	< 0.0001	< 0.0001	< 0.0001

*Data in the columns marked with the same letter of the alphabet do not differ significantly at *p* ≤ 0.05.

**Populations 5, 9, and 10 did not have any flowers.

### 3.1 Morphological characteristics of flowers

Differences were found to exist among populations in flower size, peduncle diameter, peduncle length, spathe (covering ovary), falls (sepal width), signal width, petal length and width, style arm, style crest length and width, and anther length (**Table 5**).

Among *I. hymenospatha* populations, “Arak-Rahjerd” had the largest flower size at 48.9 ± 3.0 mm, while “Ardestan-Taleghan” had the smallest at 40.2 ± 2.1 mm (**Table 5**). Among *I. hymenospatha* populations, the average peduncle length was found to be significantly higher in the “Jaro” population at 35.2 ± 1.9 mm, while the largest peduncle diameter was found in the “Abas-abad” population at 5.2 ± 0.2 mm. “Arak-Rahjerd”, “Jaro”, and “Ganjnameh” also had significant values of peduncle diameter (**Table 5**). The shortest peduncle length was found in the *I. hymenospatha* “Ganjnameh” population at 13.4 ± 2.31 mm, while the smallest peduncle diameter was found in the “Ardestan-Taleghan” population at 3.9 ± 0.3 mm (**Table 5**). Among *I. hymenospatha* populations, there was no significant difference in the spathe (covering ovary) among the “Jaro”, “Natanz-Karkas”, “Ardestan-Taleghan”, “Ganjnameh”, and “Abas-abad” populations (**Table 5**). The “Arak-Rahjerd” population had a significantly shorter spathe (9.2 ± 2.7 mm) compared to other *I. hymenospatha* populations (**Table 5**). *I. hymenospatha* “Ganjnameh”, “Jaro”, and “Abas-abad” populations had a significantly wider fall (sepal) width (11.5 ± 0.7 mm), compared to “Natanz-Karkas”, “Arak-Rahjerd”, and “Arak-Taleghan” populations (**Table 5**). In *I. hymenospatha* populations, the largest standard (petal) width was found in “Jaro” at 11.0 ± 0.4 mm and “Natanz-Karkas” at 10.7 ± 0.4 mm (**Table 5**). *I. hymenospatha* “Abas-Abad” indicated a significantly smaller style arm width and anther length among the *I. hymenospatha* population (**Table 5**). The *I. histrio* population “Velian” had a significantly larger flower size (61.3 ± 1.6 mm), plant length (27.9 ± 1.9 cm), and style crest length (11.0 ± 0.4 mm) compared to the *I. histrio* population “Ganjnameh”. The *I. histrio* population “Ganjnameh” had a significantly larger signal width between two *I. histrio* populations.

### 3.2 Morphological characteristics of bulb and leaf

Analysis of morphological variance revealed significant bulb and leaf trait diversity among *I. hymenospatha* and *I. histrio* populations (**Table 8**). Populations statistically differed in flower number, bulb diameter, bulb length, leaf number, leaf width, plant length, crown diameter, stem length under flower, and stem diameter under flower (**Table 6**). The highest number of flower among *I. hymenospatha* populations occurred in the “Arak-Rahjerd” population with approximately two flowers per accessions (**Table 6**). With regard to number of flowers, there were no significant differences among “Jaro”, “Natanz-Karkas”, “Ardestan-Taleghan”, and “Arak-Rahjerd” populations of *I. hymenospatha* (**Table 6**). The lowest number of flowers was found in the *I. histrio* “Velian” accession compared to *I. histrio* “Ganjnameh” populations (**Table 6**).

**Table 8:** Comprehensive analysis of morphological variance (AMOVA) in *Iris hymenospatha* and *Iris histrio* populations.

S.O.V.	df	Flower number	Bulb diameter	Bulb length	Leaf number	Leaf width	Plant length	Crown diameter	Stem length under flower	Stem diameter under flower
Between population	11	1.52 ^**^	29.03 ^*^	10.48 ^**^	2.30 ^**^	322.40 ^**^	78.01 ^**^	7.83 ^**^	14.49^*^	124.38^**^
Error	109	0.29	11.01	9.64	0.45	12.41	7.62	1.42	5.42	3.34

*, ** Significant difference in 5%, 1% level respectively.

The bulb diameter of *I. hymenospatha* populations ranged from 16.5 ± 1 to 20.5 ± 1 mm. The “Arsanjan” (20.5 ± 1 mm) had the highest bulb diameter and “Jaro” (29.1 ± 1 mm) had the longest bulb lengths among *I. hymenospatha* populations (**Table 6**). The bulb diameter of *I. histrio* populations ranged from 16.1 ± 1 to 18.4 ± 1 mm, but there were no significant differences among *I. histrio* populations. The *I. histrio* “Velian” population (32.1 ± 1 mm) had significantly longer bulb lengths relative to the *I. histrio* “Ganjnameh” population (21.6 ± 1 mm) (**Table 6**).

The “Arak-Gerdo”, “Abas-abad”, “Arsanjan”, and “Ganjnameh” populations had the largest number of leaves, with approximately four leaves per accession relative to other *I. hymenospatha* populations (**Table 6**). The “Ganjnameh”, “Arak-Gerdo”, and “Arsanjan” populations had the largest leaf width (20.8 ± 1.1 mm), crown diameter (8.2 ± 0.4 mm), and stem length under flower (14.0 ± 0.7 mm) among *I. hymenospatha* populations, respectively. The shortest plant length was found in the “Ardestan-Mishab” population (12.8 ± 0.9 mm) among *I. hymenospatha* populations (**Table 6**). The plant length of the *I. histrio* “Velian” population (23.1 ± 0.9 cm) was significantly longer compared to that of the *I. histrio* “Ganjnamneh” population (17.8 ± 0.9 mm) (**Table 6**).

### 3.3 Morphological characteristics of flower color and bulb tunic traits

Differences existed among *I. hymenospatha* populations in flower color, inner flower color, floral scent, bulb tunic type, leaf shape, bulb tunic color, leaf margin color, and bulb tunic density (Kruskal–Wallis, *p* < 0.0001; **Table 7**). The lavender flower color of the *I. histrio* population differed from the flower color of *I. hymenospatha*, although there was no significant difference between *I. histrio* populations (**Table 7**). In addition, large variations were observed among *I. hymenospatha* populations in flower traits, such as flower color, inner flower color, and floral scent (**Table 7**). For instance, the flower color of the *I. hymenospatha* “Abas-abad” population was significantly different among *I. hymenospatha* populations (**Table 7**). Within *I. hymenospatha* populations, flower color varied from mint cream in the “Abas-abad” population to light steel blue in the “Arak-Rahjerd” population (**Table 7**; **Figure 4**). We identified seven different inner flower colors among *I. hymenospatha* populations (**Tables 2**, **7**). *I. hymenospatha* populations “Jaro”, “Ganjnameh”, and “Abas-abad” showed substantial differences in inner flower color, with yellow-blue, yellow-white, and yellow-green colors, respectively. On the other hand, populations of *I. histrio* displayed differences inside flower color, ranging from white to purple (**Table 7**).

The flowering period of *I. hymenospatha* and *I. histrio* populations ranged from the middle of March to early April. The blooming date for the *I. hymenospatha* populations “Abas-abad” and “Ganjnameh” occurred on 29 March and 1 April 2022, respectively, which could be due to colder temperatures in the region where these accessions were located (**Table 7**). The “Velain” population of *I. histrio* had a significantly shorter flowering date compared to *I. histrio* “Ganjnameh” (**Table 7**). *I. histrio* “Velain” bloomed before March (**Table 7**). There was significant diversity in flower color, inner flower color, and tunic traits among *I. hymenospatha* and *I. histrio* populations (**Table 3**). The “Velain” population of *I. histrio* was significantly more fragrant compared to the *I. histrio* “Ganjnameh” (**Table 7**). Interestingly, the bulb tunic density of the *I. hymenospatha* “Arsanjan” was significantly higher among *I. hymenospatha* accessions (**Table 7**). Five different leaf shapes were identified among *I. hymenospatha* populations (**Table 2**; **Figure 4**). The “Ardestan-Taleghan” and “Arak-Khomain” populations of *I. hymenospatha* (long sickle-shaped leaf) showed significantly different leaf shapes compared to the *I. hymenospatha* “Arak-Gerdo” population (short erect leaf shape) (**Figure 4F**). In summary, we detected the largest flower size and number in the “Arak-Rahjerd” population of *I. hymenospatha* (**Table 5**). *I. hymenospatha* “Jaro” had the longest peduncle length, widest standard (petal) width, and longest bulb length among *I. hymenospatha* populations (**Table 5**). Moreover, the longest peduncle diameter among *I. hymenospatha* populations was in the “Abas-abad” population (5.2 ± 0.2 mm) (**Table 5**). There were no significant differences among *I. hymenospatha* populations “Abas-abad”, “Arak-Rahjerd”, and “Ganjnameh” (**Table 5**).

### 3.4 Hierarchical cluster analysis

Our use of hierarchical cluster analysis (HCA), using Ward’s method, organized *I. hymenospatha* and *I. histrio* populations (**Figure 1A**) and 120 accessions (**Figure 2A**) into five main clusters on the *y*-axis of each dendrogram. The first cluster (I) included five *I. hymenospatha* populations, which were collected from Alborz, Isfahan, and Markazi provinces, while the second cluster (II) comprised two *I. hymenospatha* populations, which were collected from Isfahan and Shiraz provinces. The third cluster (III) involved one and two populations collected from the Markazi and Hamedan provinces, respectively (**Figure 1A**). The fourth (IV) and fifth (V) clusters each included one *I. histrio* population from the Alborz and Hamedan provinces, respectively (**Figure 1A**).

The morphological traits were placed into two major clusters on the *x*-axis of each dendrogram (**Figures 1A**, **2A**). The first cluster (I) included four morphological traits: bulb length, plant length, bulb tunic type, and leaf shape (**Figure 1A**). The second cluster (II) included stem length under flower, bulb length, plant length, bulb tunic type, and leaf shape (**Figure 2A**). Morphological traits in the first cluster (I) showed minimum to moderate values in *I. hymenospatha* populations compared to the traits in the second cluster (II) (**Figures 1A**, **2A**). Most of the traits, such as flower number, leaf width, stem diameter under flower, crown diameter, bulb tunic color, leaf number, bulb diameter, stem length under flower, and bulb tunic density in the second cluster (II) indicated moderate to maximum values for *I. hymenospatha* populations compared to the first cluster (I) traits (**Figures 1A**, **2A**). Some representative morphological differences in *I. hymenospatha*, such as flower color, leaf shape, and leaf number, are shown in **Figure 4**. On the other hand, the heat map traits in the first cluster (I) for *I. histrio* populations indicated maximum values compared to the traits in the second cluster (II) on the *x*-axis of each dendrogram (**Figures 1A**, **2A**).

A constellation plot provided the best outcomes for differentiating population diversity based on the morphological traits of *I. hymenospatha* and *I. histrio* (**Figure 1B**). The constellation plot of *I. hymenospatha* and *I. histrio* populations indicated five different groups, with the same result appearing in the cluster analysis (**Figure 1A**).

The cluster analysis of morphological traits of 120 accessions demonstrated that those belonging to different populations clustered together (**Figure 2B**). The cluster analysis separated the 100 accessions of *I. hymenospatha* and 20 accessions of *I. histrio* into five groups (**Figure 2B**). Cluster I was mainly composed of *I. hymenospatha* accessions from the populations “Jaro” (32% of the population), “Arak-Khomain” (24% of the population), “Ardestan-Taleghan” (28% of the population), “Natanz-Karkas” (12% of the population), and “Ardestan-Mishab” (4% of the population) (**Figure 2B**). Cluster II consisted of *I. hymenospatha* accessions from the populations “Arak-Gerdo” (44% of the population), “Ardestan-Mishab” (25% of the population), “Arsanjan” (19% of the population), and “Natanz-Karkas” (13% of the population). Cluster III represents a combination of *I. hymenospatha* accessions from the populations of “Abas-Abad” (22.5% of the population), “Ganjnameh” (15% of the population), “Natanz-Karkas” (13% of the population), “Arsanjan” (12.5% of the population), “Arak-Gerdo” (7.5% of the population), “Ardestan-Mishab” (7.5% of the population), “Arak-Rahjerd” (5% of the population), and “Jaro” (5% of the population). Cluster IV includes accessions from the *I. histrio* population “Ganjnameh”, and cluster V consisted of the *I. histrio* population “Velian” (**Figure 2B**).

### 3.5 Principal component analysis

The arrangement of *I. hymenospatha* and *I. histrio* accessions among 10 and 2 populations, respectively, was analyzed using PCA based on morphological data (**Figure 3**). The population color and cluster number in **Figures 3A, C** are consistent with the cluster analysis shown in **Figures 2A, B**. The biplot, which visualizes the first two PCs, consistently displayed the results for 100 accessions of *I. hymenospatha* and 20 accessions of *I. histrio* (**Figures 3A, C**). These results were derived from the morphological traits that were most influential in differentiating between the accessions (**Figures 3B, D**). For *I. hymenospatha* accessions, these influential traits include stem length under flower, bulb length, plant length, leaf number, flower number, crown diameter, leaf width, and bulb diameter (**Figure 3B**).

For *I. histrio* accessions, these influential traits include leaf shape, bulb tunic color, leaf margin color, leaf width, and bulb tunic density (**Figure 3D**). The population color and cluster number are consistent with the cluster analysis shown in **Figures 2A, B**. Using the Kaiser’s criterion (“Eigenvalue” > 1) (Kaiser, 1958), two significant components were achieved, which explained 42.8% of the total variation in 100 *I. hymenospatha* accessions and 63.5% of the total variation in 20 *I. histrio* accessions (**Figure 3**). For *I. hymenospatha* accessions, PCA revealed that some traits, such as bulb length, plant length, stem length under flower, flower number, leaf width, bulb diameter, and crown diameter, had the highest scores (28.2%) relative to the total variance in the first components (**Figure 3B**). Leaf shape, bulb tunic color, bulb tunic density, and leaf number were the major contributors to the second component (PC2), which explained 16.5% of the total variance (**Figure 3B**). For *I. histrio* accessions, PCA revealed that some traits, such as leaf shape, bulb tunic color, leaf margin color, leaf width, and bulb tunic density, had the highest scores (39.6%) relative to the total variance in the first components (**Figure 3D**). Stem length under flower, crown diameter, leaf number, bulb diameter, and plant length were the major contributors to the second component (PC2), which explained 23.9% of the total variance (**Figure 3D**).

The biplot was created based on the first two PCs and mainly categorized individual accessions of *I. hymenospatha* into three independent groups (**Figure 3A**) and *I. histrio* into two independent groups (**Figure 3C**) consistent with HCA and the constellation plot (**Figures 2A, B**), with some admixture in *I. hymenospatha* populations (**Figure 3A**). Among *I. hymenospatha* populations, the second (II) and third (III) groups were plotted on the right side of PC1 (**Figure 3A**). These accessions were distinguished by bulb length, plant length, stem length under flower, flower number, leaf width, bulb diameter, and crown diameter (**Figure 3B**). Group two (II) among *I. histrio* populations was located on the right side of the PC1 (**Figure 3D**) and correlated with leaf shape, bulb tunic color, leaf margin color, leaf width, and bulb tunic density (**Figure 3D**).

## 4 Discussion

Morphological characterization is a crucial step in breeding programs as it enables the monitoring of genetic quality, allowing for the selection of the most suitable accessions for use in breeding programs. Our study shows that there are significant floral, bulb, leaf, and qualitative characteristics in *I. hymenospatha* and *I. histrio* populations collected from different regions of Iran, such as the variety of onion tunic color and onion tunic diameter. The *I. hymenospatha* populations “Arak-Rahjerd” and “Jaro” had the largest flower size (48.9 ± 3 mm) and greatest peduncle length (35.2 ± 1.9 mm), respectively (**Table 5**), which could be of value from a horticultural perspective, making these populations attractive for cultivation and conservation. Similarly, “Abas-Abad” had the largest peduncle diameter (5.2 ± 0.2 mm) among *I. hymenospatha* populations. There was no significant difference in peduncle diameter among *I. hymenospatha* “Abas-Abad”, “Arak-Rahjerd”, “Jaro”, and “Ganjnameh” populations. In our study, we observed that the populations of “Abas-Abad”, “Ganjnameh”, and “Arak-Gerdo” exhibited a similar average leaf number per accession, which was approximately 3.7. The *I. hymenospatha* “Jaro” (1.9 ± 0.2), “Natanz-Karkas” (1.7 ± 0.2), “Ardestan-Taleghan” (1.7 ± 0.2), and “Arak-Rahjerd” (2.2 ± 0.2) populations had the greatest number of flowers among the *I. hymenospatha* populations. The bulbous *Iris* inflorescence typically features two flowers, each enclosed in a spathe, which bloom in sequence (Kamenetsky and Okubo, 2012). The *I. histrio* “Velian” population had a significantly higher flower size and peduncle length, relative to the *I. histrio* “Ganjnameh” population. These findings highlight the potential of horticulture in conserving these species. By selecting for these traits in cultivation, we can help in the conservation of these species.

Morphological characterization is a crucial step in breeding programs as it enables the monitoring of genetic quality, allowing for the selection of the most suitable accessions for use in breeding programs. This is particularly important for species like *I. hymenospatha* and *I. histrio*, which are facing threats in their native habitats. Horticulture can play a significant role in the conservation of such species by preserving their genetic diversity and promoting their propagation. Our study shows that there is significant floral, bulb, leaf, and qualitative characteristics in *I. hymenospatha* and *I. histrio* populations collected from different regions of Iran, such as the variety of onion tunic color and onion tunic diameter. These traits not only have horticultural value, but also contribute to the species adaptability and survival.

Our results are consistent with those of Moradi et al. (2023), who investigated morphological variability in wild-growing, but threatened *Fritillaria imperialis* populations (Moradi et al., 2023). They displayed several key ornamental traits that are highly suitable for breeding initiatives, such as plant length, peduncle length, peduncle diameter, flower number, flower size, and leaf number (Moradi et al., 2023). Wild-growing populations of *F. imperialis* are rapidly declining due to overgrazing, overharvesting, and climate change. Similar to our results, Khaleghi and Khadivi (2022) noted that flower characteristics, such as inflorescence length, diameter, and color; floral scent; and peduncle length, are of great ornamental and commercial importance.

In this study, we found that the *I. hymenospatha* population “Arak-Rahjerd” has a light steel blue flower color, which is significantly different from the mint cream color found in the “Abas-abad” population (**Table 7**; **Figure 4**). Flower color is one of the most important concerns for breeders due to its influence on ornamental and commercial value (Nakamura et al., 2015). Members of the *Iris* genus display a wide variety of flower colors, including dark purple, violet, pink, yellow, and white flowers (Roguz et al., 2020). Owing to distinctive features, such as the fragrance and color variety of *I. hymenospatha*, they can be used as breeding stock for new cultivars (Asgari et al., 2022). Given the aesthetic quality of the flowers of *I. hymenospatha*, they exhibit potential to be introduced and further developed as ornamental flowers (Asgari et al., 2022). Various traits in this bulbous species underscore how it can be used to select desired attributes for development, efficiency, and commercial development.

The *I. histrio* “Velian” population had a significantly higher flower size, floral scent, peduncle length, style arm width, flowering date, and plant length compared to the “Ganjnameh” population of *I. histrio*. From an ornamental and conservation perspective, the scent of a flower is of great importance. In addition, flower scent and color may be derived from the same biosynthesis pathways (Roguz et al., 2020). Morphological characteristics of qualitative traits showed significantly shorter flowering date of the *I. hymenospatha* “Ganjnameh” and “Abas-Abad” populations, which bloomed on 1 April and 29 March, respectively (**Table 5**). The *I. hymenospatha* “Jaro” (11.0 ± 0.4 mm) and “Natanz-Karkas” (10.7 ± 0.4 mm) populations had the widest standard (petal) width among populations (**Table 3**). A significantly higher signal width was reported in *I. hymenospatha* “Jaro” (5.1 ± 0.2 mm) and “Ganjnameh” (5.2 ± 0.3 mm) among *I. hymenospatha* populations (**Table 5**). Similar to our results, Azimi et al. (2016) collected 14 *Iris* species from different provinces of Iran and classified them into three clusters based on quantitative traits and four groups based on qualitative traits. According to Azimi et al. (2016), the components of *Iris* flowers that have significant economic implications for breeding programs include the quantitative traits of flower size, outer and inner tepal width, inner tepal length, and leaf width.

In our study, characteristics such as bulb length, plant length, stem length under flower, flower number, leaf width, bulb diameter, and crown diameter contributed to the first component (PC1) and leaf shape, bulb tunic color, bulb tunic density, and leaf number were the major contributors to the second component (PC2) in the biplot analysis of the two *Iris* species. With 42.8%, the first two components had the highest scores relative to the total variance in *I. hymenospatha* (**Figures 3A, B**). Our PCA findings show that morphological characteristics, such as the stem length under flower, bulb length, plant length, leaf number, flower number, crown diameter, leaf width, and bulb diameter, are the most relevant morphological traits in *I. hymenospatha* (PC1) (**Figure 3B**). To summarize, the accessions of *I. hymenospatha* from the “Arak-Rahjerd”, “Ganjnameh”, “Arak-Gerdo”, and “Abas-abad” populations have the highest number of these traits (PC1) (**Figure 3A**). Out of all the *I. hymenospatha* populations, they appear to have the greatest potential for selection in breeding programs (**Tables 5**, **6**).

Leaf shape, bulb tunic color, leaf margin color, leaf width, stem length under flower, crown diameter, leaf number, and bulb diameter emerge as primary contributors to the first component (PC1) in the populations of *I. histrio* we analyzed. Plant length, bulb length, and flower number are identified as major contributors to the second component (PC2) (**Figures 3C, D**). These components collectively explain 63.5% of the total variance (**Figures 3C, D**). In summary, the accessions of *I. histrio* from the “Ganjnameh” populations have the highest number of these relevant traits (PC1) (**Figures 3C, D**). *I. histrio* “Ganjnameh” populations collected from the province of Hamedan (**Table 1**) appear to have the greatest potential for selection as an ornamental bulbous crop.

PCA has been previously used to evaluate the genetic relationship of other plant species, such as *Muscari* (Labbaf et al., 2020) and *Tulipa* (Khaleghi and Khadivi, 2022). Azimi et al. (2018) investigated the morphological characteristics of *Iris germanica* hybrids and found a 97% heritability value for plant height, fall width and length, flower size, and leaf width. They concluded that these values can be used as useful traits in *Iris* breeding and hybrid selection. Asgari et al. (2022) studied five *Iris* species and derived four factors from multivariate morphological analysis that explained 84.79% of total variance. Consistent with our results, Asgari et al. (2022) reported that some variables, such as petal length, flower height, seedling number, number of flowers, bulb length, leaf number, bulb diameter, leaf width, and stem diameter, were significant in explaining variation among accessions.

## 5 Conclusion

Populations of *I. hymenospatha* from “Jaro”, “Natanz-Karkas”, “Ardestan-Taleghan”, “Arak-Rahjerd”, “Arak-Gerdo”, “Ganjnameh”, and “Abas-Abad”, which belong to clusters I and III (**Figures 1**, **2**, and **3A**), exhibited moderate to maximum values in selected traits such as flower number, leaf width, stem diameter under flower, crown diameter, bulb tunic color, leaf number, bulb diameter, stem length under flower, and bulb tunic density (**Figure 3B**). These traits make these populations not only valuable for horticultural purposes but also crucial for conservation efforts. The use of horticulture as a tool for conservation is particularly important for these populations, as it allows for the preservation of their genetic diversity and resilience. These traits appear useful for further selection of *I. hymenospatha* accessions for ornamental purposes. Moreover, the *I. histrio* “Velian” population had a significantly higher flower size, peduncle length, style arm width, flowering date, and plant and bulb length compared to the “Ganjnameh” population of *I. histrio.* Variation in such traits suggests that this species should be further evaluated for horticultural purposes (**Figures 1A**, **2A**).

Our results indicate that the five populations of *I. hymenospatha* (“Arak-Rahjerd”, “Arak-Gerdo”, “Jaro”, “Abas-Abad”, and “Ganjnameh”), which we collected from Alborz, Markazi, and Hamedan provinces, showed wide variation in morphological traits, such as flower size, peduncle diameter, peduncle length, flower number, leaf number, flower color, and flower scent. As such, they can be used in breeding programs to improve commercial cultivars (**Tables 5**–**7**). In addition, some accessions of “Ganjnameh” and “Abas-abad” had contractile roots that are responsible for the movement of the underground portion of the species (I. Rohollali, personal observation). On the other hand, from an ornamental point of view, the “Arak-Rahjerd”, “Ganjnameh”, and “Abas-Abad” populations of *I. hymenospatha* seem to be the most promising for conservation breeding programs since they were characterized by a wide variation of horticulturally important qualitative traits such as flower color and flowering date (**Table 7** and **Figure 4**).

Global biodiversity is declining at an unprecedented rate (12.5% of the estimated world flora), and immediate conservation action is required to protect many of these species (Walter and Gillett, 1998). The collection of plant material in this study was carried out in accordance with the rules of the Genetic Resources Protection and Exploitation Law of the Government of Iran. We stored the collected seeds of both *Iris* species in the gene bank of Shahed University in Tehran, Iran. All relevant information regarding the preservation of the living germplasm of both species will be donated to the Iranian Biological Resource Center.

Taken together, we confirmed the hypothesis that there is broad morphological variation among *I. hymenospatha* and *I. histrio* accessions in Iran at the population scale. We determined several morphological characters that can be used to introduce *I. hymenospatha* and *I. histrio* into conservation and breeding programs for further development as horticultural selections. Protection of scattered habitats, even those relatively small in size, including small populations of *I. hymenospatha* and *I. histrio*, which naturally thrive in such semi-arid and arid regions, is of great importance. *I. hymenospatha* populations naturally grow in regions with low rainfall such as Isfahan, where the “Ardestan-Taleghan” population was located, and in regions with high rainfall, such as Hamedan, where the “Ganjnameh” and “Abas-abad” populations occurred (**Table 2**). The species also grows in a variety of soils, from rocky to sandy clay, between altitudes of 1,655 and 2,212 m with reasonable adaptability to a variety of natural conditions. Conserving these species while allowing for sustainable ornamental use of these species should continue to be pursued.

## Data availability statement

The datasets presented in this study can be found in online repositories. The names of the repository/repositories and accession number(s) can be found below: https://www.try-db.org/TryWeb/Data.php#101.

## Author contributions

IR: Conceptualization, Funding acquisition, Methodology, Project administration, Resources, Writing – original draft. AN: Writing – review & editing, Data curation, Formal analysis, Supervision. JS: Writing – review & editing, Funding acquisition. RK: Investigation, Writing – review and editing.
