# Supplementary material for: Morphological characterization of Iris hymenospatha and Iris histrio populations in Iran: implications for conservation and breeding
Source: Front Plant Sci. 2024 May 28;15:1305240. doi: 10.3389/fpls.2024.1305240 (PMC11165347; doi:10.3389/fpls.2024.1305240)
Supplement: Supplementary file 1 [file Table_1.docx]

Supplementary Material

# Supplementary Tables

## Supplementary Table 1. Morphological characteristics correlation of *I. hymenospatha* and *I. histrio* populations.

| Row | FN | BD | BL | LN | LW | PL | CD | SLF | SDF | BTT | LS | BTC | BTD |
| --- | --- | --- | --- | --- | --- | --- | --- | --- | --- | --- | --- | --- | --- |
| FN^*^ | 1.00 |  |  |  |  |  |  |  |  |  |  |  |  |
| BD | -0.13 | 1.00 |  |  |  |  |  |  |  |  |  |  |  |
| BL | -0.18 | -0.06 | 1.00 |  |  |  |  |  |  |  |  |  |  |
| LN | 0.46^*^ | 0.29^**^ | -0.25^*^ | 1.00 |  |  |  |  |  |  |  |  |  |
| LW | 0.71^**^ | 0.37^**^ | -0.01 | 0.53^**^ | 1.00 |  |  |  |  |  |  |  |  |
| PL | -0.41^*^ | -0.16 | 0.65^**^ | -0.16 | -0.52^*^ | 1.00 |  |  |  |  |  |  |  |
| CD | 0.48^**^ | 0.42^**^ | -0.22^*^ | 0.41^**^ | 0.71^**^ | -0.62^**^ | 1.00 |  |  |  |  |  |  |
| SLF | 0.29^*^ | 0.43^**^ | -0.21^*^ | 0.28^*^ | 0.23 | 0.11 | 0.09 | 1.00 |  |  |  |  |  |
| SDF | 0.51^**^ | 0.50^**^ | 0.17 | 0.52^**^ | 0.92^**^ | -0.32^*^ | 0.56^**^ | 0.20 | 1.00 |  |  |  |  |
| BTT | -0.73^**^ | -0.27 | 0.14 | -0.48^**^ | -0.90^**^ | 0.66^**^ | -0.86^**^ | -0.14 | -0.71 | 1.00 |  |  |  |
| LS | 0.14 | -0.17 | 0.01 | -0.57^**^ | -0.10 | 0.06 | -0.06 | 0.09 | -0.14 | 0.16 | 1.00 |  |  |
| BTC | 0.57^**^ | 0.45^**^ | -0.47^**^ | 0.30^*^ | 0.75^**^ | -0.70^**^ | 0.66^**^ | 0.47^**^ | 0.58^**^ | -0.75^**^ | 0.20 | 1.00 |  |
| BTD | 0.30^*^ | 0.20 | -0.37^**^ | 0.04 | 0.18 | -0.57^*^ | 0.45^*^ | 0.23^*^ | 0.01 | -0.45^*^ | -0.24 | 0.35^*^ | 1.00 |

*Abbreviation mentioned in Table 2 (Morphological characters and their abbreviations).
